# Prevalence and predictors of post-COVID-19 symptoms in general practice - a registry-based nationwide study

**DOI:** 10.1186/s12879-023-08727-6

**Published:** 2023-10-25

**Authors:** Øystein Hetlevik, Knut-Arne Wensaas, Valborg Baste, Knut Erik Emberland, Türküler Özgümüs, Siri Eldevik Håberg, Guri Rortveit

**Affiliations:** 1 Department of Global Public Health and Primary Care, University of Bergen, Postbox 7804, Bergen, NO-5020 Norway; 2 Research Unit for General Practice, NORCE Norwegian Research Centre, Bergen, Norway; 3 National Centre for Emergency Primary Health Care, NORCE Norwegian Research Centre, Bergen, Norway; 4 Centre for Fertility and Health, The Norwegian Institute of Public Health, Oslo, Norway

**Keywords:** Post-COVID-19, SARS-CoV-2, General practice, Risk factors, Symptoms, Registry-based study, Nationwide

## Abstract

**Background:**

With Norwegian national registry data, we assessed the prevalence of post-COVID-19 symptoms at least 3 months after confirmed infection, and whether sociodemographic factors and pre-pandemic health problems were risk factors for these symptoms.

**Methods:**

All persons with a positive SARS-CoV-2 PCR test from February 2020 to February 2021 (exposed) were compared to a group without a positive test (unexposed) matched on age, sex, and country of origin. We used Cox regression to estimate hazard ratios (HR) for 18 outcome symptoms commonly described as post-COVID-19 related, registered by GPs. We compared relative risks (RR) for fatigue, memory disturbance, or shortness of breath among exposed and unexposed using Poisson regression models, assessing sex, age, education, country of origin, and pre-pandemic presence of the same symptom and comorbidity as possible risk factors, with additional analyses to assess hospitalisation for COVID-19 as a risk factor among exposed.

**Results:**

The exposed group (N = 53 846) had a higher prevalence of most outcome symptoms compared to the unexposed (N = 485 757), with the highest risk for shortness of breath (HR 2.75; 95%CI 2.59–2.93), fatigue (2.08; 2.00-2.16) and memory disturbance (1.41;1.26–1.59). High HRs were also found for disturbance of smell/taste and hair loss, but frequencies were low. Concerning risk factors, sociodemographic factors were at large similarly associated with outcome symptoms in both groups. Registration of the outcome symptom before the pandemic increased the risk for fatigue, memory disturbance and shortness of breath after COVID-19, but these associations were weaker among exposed. Comorbidity was not associated with fatigue and shortness of breath in the COVID-19 group. For memory disturbance, the RR was slightly increased with the higher comorbidity score both among exposed and unexposed.

**Conclusion:**

COVID-19 was associated with a range of symptoms lasting more than three months after the infection.

**Supplementary Information:**

The online version contains supplementary material available at 10.1186/s12879-023-08727-6.

## Background

Since the start of the COVID-19 pandemic in 2020, patients have reported various long-term symptoms after infection, and the term “Long-COVID” was introduced in May 2020 by patients’ initiatives [1]. The variety of symptoms associated with Long-COVID caught widespread interest, not least as a challenge for primary care, as summarised in a “Primer for family physicians” in the autumn of 2020 [2]. During the pandemic, the scientific literature on long-term health problems following COVID-19 have been growing rapidly [3–6].

Several terms and definitions have been proposed for health problems following COVID-19 [7]. The WHO definition from 2021 used the term “post-COVID-19” for symptoms from 3 months after the onset of COVID-19 [8]. The British Institute for Health and Care Excellence (NICE) recommends the term “Post-COVID-19 syndrome” for persistent or new signs and symptoms present more than 12 weeks after acute COVID-19 [9].

Post-infectious health problems are well-documented for a variety of infectious diseases, but the mechanisms by which the acute infection leads to later symptoms are unclear [10]. A particular challenge is to differentiate between generic post-infectious symptoms that are common after many infectious diseases, and possible long-term symptoms specific to COVID-19 infection. It is possible that COVID-19 can give widespread affection of different organs through inflammatory and immunological mechanisms [11], explaining the variance in the suggested symptoms of post-COVID-19 syndrome.

Early reviews on symptoms following COVID-19 reported that the most frequent persistent symptoms occurring one to six months after COVID-19 were fatigue, dyspnoea, arthralgia, headache, sleep disturbance and mental health problems, concentration difficulties, and memory loss. A meta-analysis in 2022 including 43 studies of hospitalized and non-hospitalized patients with follow-up time ranging from 30 to 120 days found the pooled prevalence of persistent symptoms to be 43%, with moderate variations between geographic regions [12]. Later, a meta-analysis in 2023 based on studies with a longer observation period reported the prevalence of post-COVID-19 symptoms 12 months after the infection to be below 1% among non-hospitalised, and 11% in hospitalised patients, doubling the prevalence if the stay included intensive care [6]. Higher age, female sex, comorbidity, the severity of the COVID-19 infection, and in some studies also sociodemographic factors, are found to be risk factors for post-COVID-19 symptoms [4, 5, 13].

Symptoms currently identified as post-COVID-19 are frequent in the general population without prior infection. There is a lack of studies from primary care addressing whether such symptoms occur more frequently among people who have had COVID-19 than in the general population. Also, we do not know whether risk factors for these common symptoms differ between people who have had COVID-19 compared to those without a positive SARS-CoV-2 PCR test.

We used nationwide data from Norwegian primary care and designed a study with the following aims: (1) To compare the prevalence of possible post-COVID-19 symptoms (outcome symptoms) registered by primary care physicians at least 3 months after confirmed COVID-19 (exposed) to the prevalence in a matched control population without positive SARS-CoV-2 PCR test (unexposed). (2) To investigate if risk factors such as sociodemographic factors, prior illness/comorbidity, or pre-pandemic presence of symptoms acted differently for selected outcome symptoms after COVID-19 as compared to an unexposed group.

## Methods

This is a prospective population-based cohort study based on nationwide registry data from Norway in the period 2019–2021. The population at risk included all residents in Norway 18 years and above in 2020 who were not living in a nursing home before February 2020 (N = 4 278 768). Exposure-density sampling is used to sample unexposed controls for each exposed persons from the population at risk.

### Study context

Norway has a public health care system with universal health coverage. All citizens are assigned to a specific general practitioner (GP), who is responsible for the management of acute and chronic disease including follow-up of symptoms after acute illness. Emergency out-of-hours services are run by the municipalities and staffed with GPs on rotation. Specialist care and hospitalization require referral from a primary care physician or direct admission by ambulance. During the pandemic public test-centres were set up where people could get tested free of charge. In Norway most COVID-19 patients were evaluated and treated in primary care.

### Data sources

All SARS-CoV-2 PCR test results were mandatorily reported to a registry run by the Norwegian Surveillance System for Infectious Diseases (MSIS) [14], and from there we retrieved positive PCR test results and the test dates. The Norwegian Registry for Primary Health Care (NRPHC) include data from reimbursement claims submitted by GPs and municipalities and contains information about all contacts with GPs or other primary care physicians in day-time practice and the out-of-hours services [15]. All GP contacts include diagnostic information according to International Classification of Primary Care (ICPC-2). These codes were used to determine the pre- and post-COVID symptom diagnoses and registrations of chronic diseases prior to the pandemic. From NRPHC we also captured information on whether persons are residents in a nursing home since these patients are not served by GPs and no diagnoses are available in registers. The Norwegian Patient Registry (NPR) contains data from secondary care, including both outpatient clinics and hospitals. NPR was used to identify COVID-19 related hospital admissions. Norwegian Intensive Care and Pandemic Registry data was retrieved to determine COVID-19 related stays in intensive care. Norwegian Cause of Death Registry provided information on date of death for residents in Norway. Sociodemographic data for the study participants were retrieved from Statistics Norway.

Using the national identifier that is unique for all residents, information from all registries were linked at an individual level. New project specific identifiers for each person were generated and personal identification numbers were removed before the datafiles were sent to the researchers.

### Study population

**Fig. 1:**
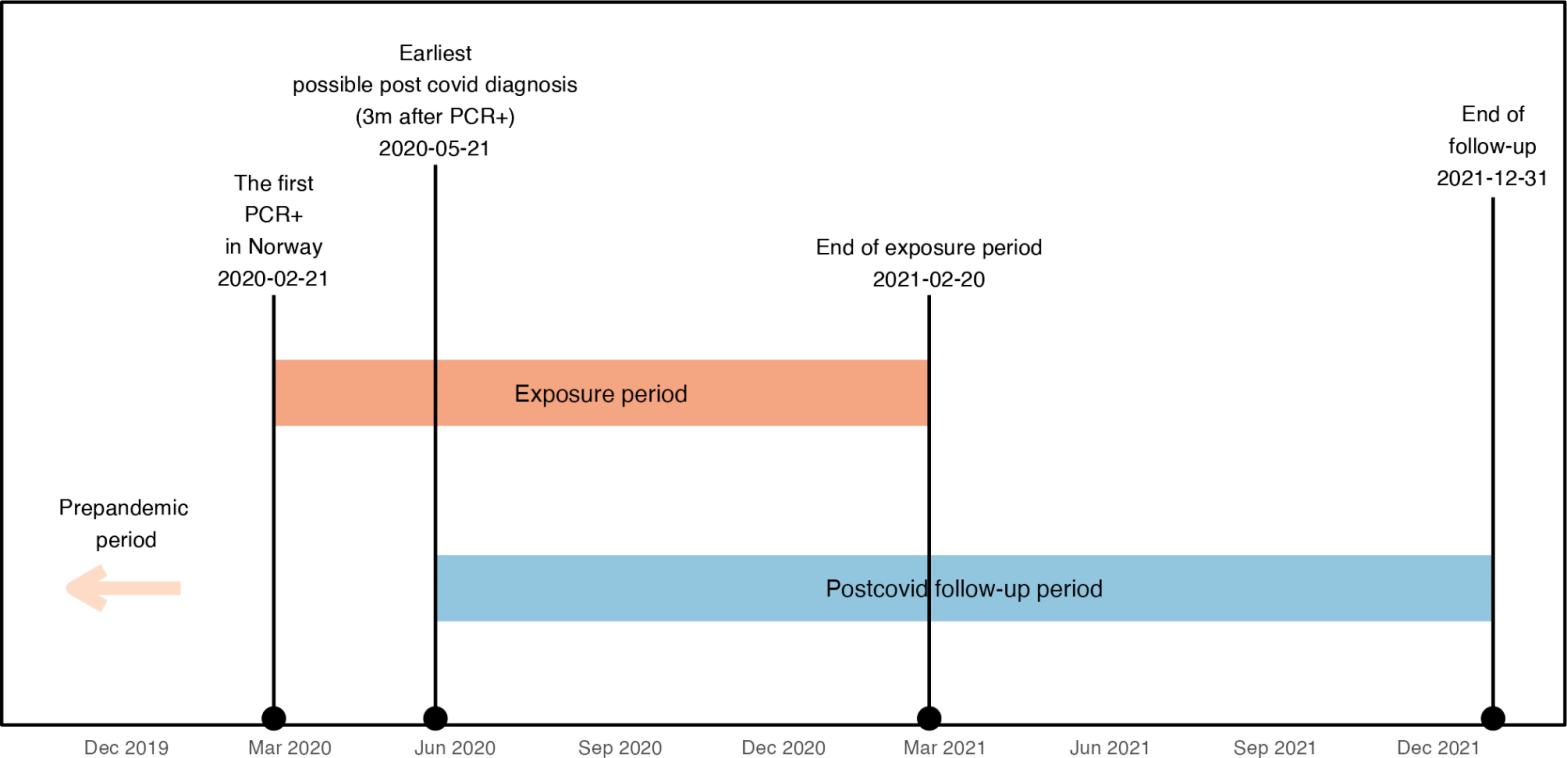
Timeline for the study period. Patients were included between 21 February 2020 and 20 February 2021, and outcome symptoms registered from 21 May 2020 until 31 December 2021, but maximally 12 months after inclusion

All persons with a positive PCR test for SARS-CoV-2 between 21 February 2020 and 20 February 2021 were included in a COVID-19 group and denoted as “exposed” (Fig. 1).

**Fig. 2:**
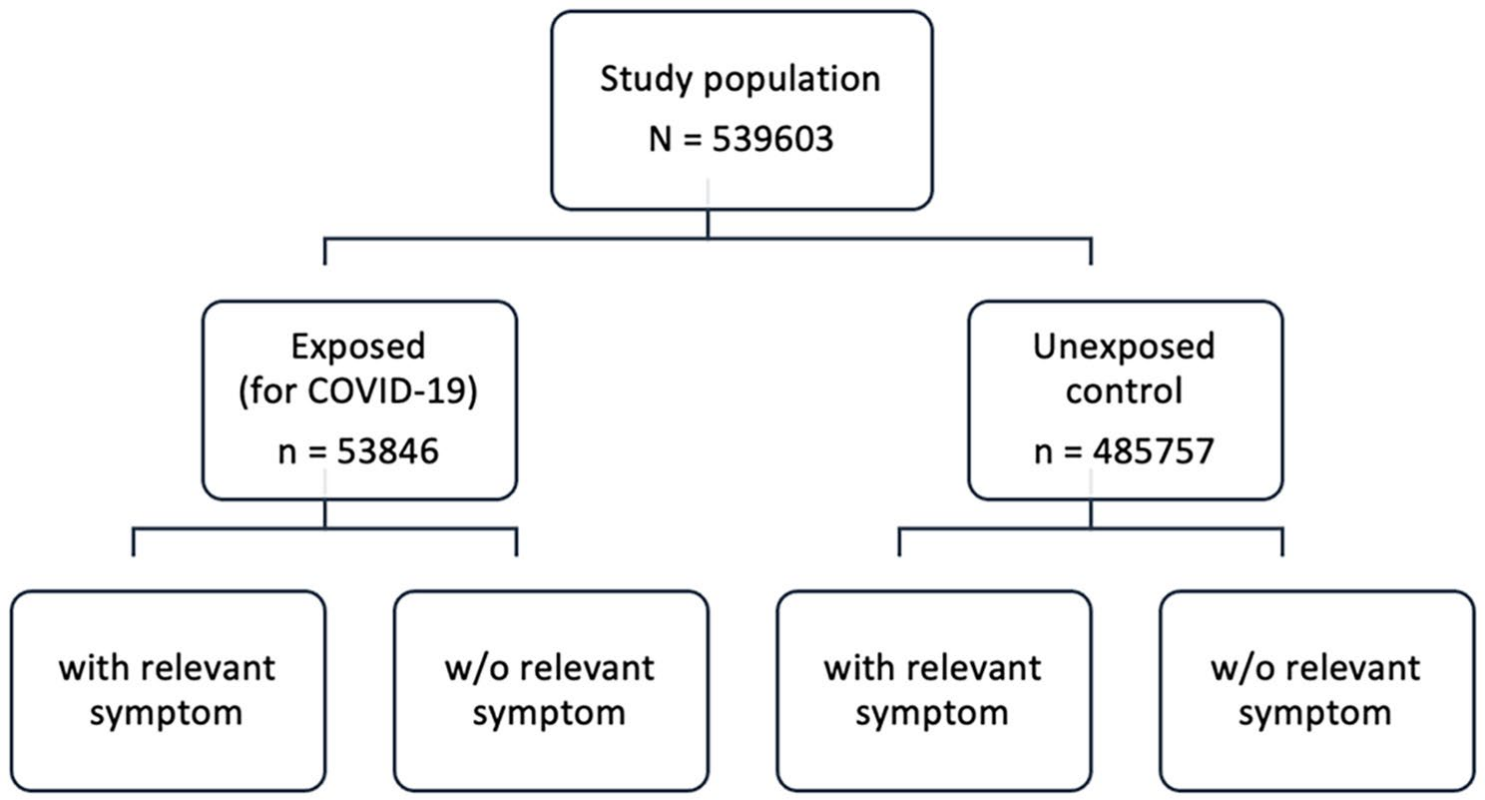
Overview of the study population. Based on nationwide register data we identified all persons in Norway who tested positive for COVID-19 in Norway during first year of the pandemic, defined as exposed. For each exposed, 10 matched persons without positive COVID-19 PCR test (unexposed controls) were selected. Exposed and unexposed controls were further divided into those with and without any symptom of interest in further analyses

Further, exposure density sampling was used to select a comparison group of controls without positive COVID-19 PCR test [16]. Selection of the unexposed controls was done randomly with matching for age, sex, and country of origin. For each exposed person 10 random controls were selected among persons who had no positive PCR test before sampling and were under risk of having a positive test at the time point of matching. Due to random and separate sampling of controls for each person with a positive PCR test, some controls were sampled several times for different exposed persons. However, in the final dataset a control is included only once to avoid duplication. Therefore, the size of the final control group is smaller than what is expected with 1:10 ratio of exposed to unexposed controls. The study population comprised 539 603 patients (Fig. 2).

### Exposure, outcome symptoms and covariates

The exposure was having had COVID-19 defined as a positive PCR test for SARS-CoV-2 between 21 and 2020 and 20 February 2021.

Outcome variables were diagnoses commonly included as post-COVID-19 symptoms in previous studies [12, 17–19]. The ICPC-2 codes used to define the outcome symptoms are shown in Table 1. We collected diagnoses from all GP consultations in the period from 3 to 12 months after a positive PCR, and in the corresponding period for matched unexposed controls. The first eligible date for registration of outcome symptoms was 21 May 2020.

**Table 1:** Selected post-COVID-19 symptoms (outcome symptoms) and the corresponding ICPC-2 codes from general practice consultations

Outcome symptoms	ICPC-2 codes
Fatigue	A04 Weakness/tiredness general
Chest pain	A11 Chest pain
Abdominal pain	D01 Abdominal pain/cramps general D02 Abdominal pain epigastric
Palpitations	K04 Palpitations
Myalgia	L01 Neck symptom/complaint L02 Back symptom/complaint L03 Low back symptom/complaint L04 Chest symptom/complaint L09 Arm symptom/complaint L14 Leg/thigh symptom/complaint L18 Muscle pain L19 Muscle symptom/complaint NOS L29 Musculoskeletal symptom/complaint other
Arthralgia	L08 Shoulder symptom/complaint L09 Arm symptom/complaint L10 Elbow symptom/complaint L11 Wrist symptom/complaint L12 Hand/finger symptom/complaint L13 Hip symptom/complaint L15 Knee symptom/complaint L16 Ankle symptom/complaint L17 Foot/toe symptom/complaint L20 Joint symptom/complaint NOS
Headache	N01 Headache
Disturbance of smell taste	N16 Disturbance of smell/taste
Dizziness	N17 Dizziness
Sleep disturbance	P06 Sleep disturbance
Memory disturbance	P20 Memory disturbance
Anxiety	P74 Anxiety
Depression	P76 Depression
Psychological symptoms	P01 Feeling anxious/nervous/tense, P02 Acute stress reaction P03 Feeling depressed P04 Feeling/behaving irritable/angry
Shortness of breath	R02 Shortness of breath
Cough	R05 Cough
Throat symptoms	R21 Throat symptoms
Hair loss	S23 Hair loss

We used the following covariates: sex, age (categorical, < 25, 25–40, 41–60 and > 60), highest fulfilled education in 2019 (4 categories as no education, elementary school, high school and higher education), country of origin (6 categories as Norway, Europe, Africa, North America and Oceania, Asia, South and Central America), pre-pandemic registration (21 February 2019 to 20 February 2020) of the same symptom as the outcome symptom, the number of pre-pandemic chronic conditions selected among a list of diagnoses defined by ICPC-2 codes as shown in Supplementary file Table S1 (4 categories as 0, 1, 2, 3 or more conditions) [20] and pre-pandemic number of GP consultations (categorical, 0, 1–2, 3–5 and 5+).

Hospitalization due to COVID-19 was defined as registration of one of ICD-10 codes U071, U072, B342, B972 in NPR with more than 5 h of hospital stay during the exposure period.

### Statistical analysis

To investigate the risk for different outcome symptoms after COVID-19 we applied Cox regression models with a positive SARS-CoV-2 PCR test as a time-dependent exposure variable to obtain hazard ratios (HRs) with 95% confidence interval (CI) for each outcome symptom. Cluster robust standard errors were used to account for clustering of individuals who were included in the study population first as unexposed but later had a positive PCR test and included as exposed. Other covariates were education, pre-pandemic registration of the same symptom as the outcome symptom, pre-pandemic registration comorbidities, and pre-pandemic number of consultations.

The follow-up periods were calculated separately for each of the 18 diagnoses listed in Tables 1 and 18 separate regression models were used. The exposed persons were followed up from 3 months after their positive PCR test date to an event (outcome symptom). Unexposed controls were followed up from 3 months after they were sampled to the date of an event (outcome symptom) (Fig. 1).

An unexposed control was censored at the time of the PCR test if he/she had a positive test. If the positive PCR test was before 20 February 2021, which is the latest test date to be included in as exposed, the person was transferred to the exposed group, contributing with time as unexposed from sampling time to a positive test, and with time as exposed from 3 months after the positive test.

Persons were also censored 12 months after their sampling date or at end of follow-up on 31 December 2021, at day of emigration or death, or if they moved to a nursing home during the study period. Patients treated for severe COVID-19 in intensive care were censored at their date of entry to intensive care unit due to an overlap between symptoms commonly found after intensive care in general and possible post-COVID-19 symptoms.

HRs were calculated by using above explained models including the adjustment variables and are presented with their 95% confidence intervals in the tables and in the text.

Secondly, to investigate possible risk factors for the post-COVID-19 symptoms fatigue, memory disturbance and shortness of breath we used separate Poisson regression models with robust standard errors to estimate RRs. The three outcome symptoms were chosen as markers for different clusters of previously described post-COVID symptoms such as fatigue (with bodily pain or mood swings), cognitive problems and ongoing respiratory problems [6]. We used the following predictor variables: sex, age, education, country of origin, pre-pandemic registration of the same symptom as the outcome symptom and comorbidities. The pre-pandemic number of GP consultations was added as covariate in these models. The analyses were performed separately for exposed and unexposed controls (stratification by exposure status) (Fig. 2). The prevalence and adjusted RR estimates with 95% CI are presented in plots stratified by exposed/unexposed status and in Supplementary file Tables S2, S3, S4. For the exposed group we also had a similar model where hospitalization due to COVID-19 was added as predictor as an indicator for severeness of infection presented in Supplementary file Table S5 for all outcome symptoms.

Further, we investigated whether the possible risk factors acted differently for those with COVID-19 compared to the unexposed control, and tested whether there was an interaction between the exposure (COVID-19) and each predictor variable (risk factor). We applied multivariable modified Poisson models (including all other variables) with an interaction term between COVID-19 yes/no and the predictor (one-by-one) for each outcome. The p-values of interaction terms are presented in Supplementary file Tables S2b, S3b and S4b. Due to multiple testing a significant level of α = 0.001 was considered statistically significant for the interaction tests. In other analyses we used a 95% confidence interval to investigate statistical differences.

## Results

We identified 53 846 persons aged 18 to 102 years with a positive SARS-CoV-2 PCR test in the study period. The mean age was 41 years and 51.6% were male (Table 2). Half of the sample were born in Norway, 18.8% in other European countries, 18.2% in Asia and 10.8% in Africa. The exposed and unexposed group were well matched for most characteristics, but a slightly higher proportion of unexposed were born in Norway (52.3% vs. 49.6% of exposed).

**Table 2:** Descriptive characteristics of the study population

	Exposed group (Pos PCR for SARS-CoV-2)		Unexposed group (Matched controls)	
	N	%	N	%
**Total number**	53,846		485,757	
**Sex**, *male*	27,800	51.6	250,045	51.5
**Age** *(years), mean (SD)*	41.02	(16.07)	41.56	(16.26)
**Education (highest registered)**				
*No education registered*	3531	6.6	30,430	6.3
*Primary education*	15,285	28.4	137,694	28.3
*High school*	16,956	31.5	155,724	32.1
*Higher education*	18,074	33.6	161,909	33.3
**Origin of country**				
*Norway*	26,720	49.6	254,054	52.3
*Europe*	10,125	18.8	92,008	18.9
*Africa*	5445	10.1	41,702	8.6
*North America and Oceania*	1426	2.6	13,211	2.7
*Asia*	9806	18.2	81,894	16.9
*South and Central America*	324	0.6	2888	0.6
**GP consultations in 2019**				
*Number consultations, mean (SD)*	2.49	(3.65)	2.35	(3.59)
*Proportion with* ≥ *1 consultation*		64.6		61.8
**Number of comorbid conditions** ^**a**^				
*0*	44,474	82.6	399,623	82.3
*1*	7587	14.1	69,572	14.3
*2*	1540	2.9	13,994	2.9
*3+*	245	0.4	2568	0.5
**Hospitalisation due to COVID-19** ^**b**^	2522	4.7	0	0

a) based on a selection of the diagnosis from GPs in 2019 (supplementary file, table S1)

b) hospitalised with acute Infections (those with a stay in intensive care units were excluded)

### Prevalence of post-COVID-19 symptoms in general practice

The exposed and the unexposed groups had similar prevalence of the selected outcome symptoms in 2019 (Table 3).

**Table 3:** Selected outcome symptoms registered in Norwegian general practice in 2019 and 3 to 12 months after positive SARS-CoV-2 PCR or inclusion as controls

	Pre-COVID period (in 2019)				3–12 months after a SARS-CoV-2 PCR test/inclusion					
	Exposed group		Unexposed controls		Exposed group		Unexposed controls		HR*	**95% CI**
	N	%	N	%	N	%	N	%		
**Outcome symptoms**										
Fatigue	1686	3.1	15,071	3.1	3226	6.0	14,206	2.9	2.08	2.00-2.16
Chest pain	426	0.8	3294	0.7	802	1.5	5552	1.1	1.30	1.20–1.40
Abdominal pain	2428	4.5	19,386	4.0	2360	4.4	17,196	3.5	1.22	1.17–1.28
Palpitations	480	0.9	4169	0.9	636	1.2	4731	1.0	1.19	1.09–1.29
Myalgia	4409	8.2	34,114	7.0	5361	10.0	40,429	8.3	1.15	1.12–1.19
Arthralgia	4331	8.0	36,869	7.6	5333	9.9	43,757	9.0	1.07	1.04–1.11
Headache	1400	2.6	10,110	2.1	1502	2.8	9485	2.0	1.39	1.31–1.47
Disturbance of smell/taste	15	< 0.1	150	< 0.1	226	0.4	137	< 0.1	15.17	12.23–18.83
Dizziness	827	1.5	7108	1.5	932	1.7	7003	1.4	1.18	1.10–1.27
Sleep disturbance	1855	3.4	16,528	3.4	1517	2.8	14,666	3.0	0.94	0.89–0.99
Memory disturbance	873	1.6	7964	1.6	341	0.6	2171	0.4	1.41	1.26–1.59
Anxiety	156	0.3	1315	0.3	598	1.1	6122	1.3	0.90	0.83–0.99
Depression	584	1.1	5999	1.2	1656	3.1	16,135	3.3	0.92	0.88–0.97
Psychological symptoms	1723	3.2	16,715	3.4	2358	4.4	20,432	4.2	1.03	0.99–1.08
Shortness of breath	525	1.0	4553	0.9	1426	2.6	4760	1.0	2.75	2.59–2.93
Cough	1431	2.7	11,489	2.4	1056	2.0	7556	1.6	1.20	1.13–1.29
Throat symptoms	844	1.6	6641	1.4	775	1.4	5417	1.1	1.27	1.17–1.37
Hair loss	153	0.3	1115	0.2	241	0.4	1097	0.2	1.92	1.66–2.22

Number, proportion of patients with selected outcome symptoms registered in Norwegian general practice in 2019 and 3 to 12 months after positive SARS-CoV-2 PCR test (exposed, N = 53 846) or inclusion as unexposed control (N = 485 757). Adjusted hazard ratio (HR) with 95% confidence interval (CI) for outcome symptoms among exposed compared to unexposed 3–12 months after a positive PCR test/inclusion

* Education, pre-pandemic registration of the same code(s) as outcome symptom, comorbidities, and pre-pandemic number of consultations (all defined in 2019) were included as time-constant covariates

The exposed had a higher probability of being registered with most outcome symptoms 3 to 12 months after COVID-19 compared to the unexposed, especially with fatigue (HR 2.08; 95% CI 2.00-2.16), shortness of breath (HR 2.75; CI 2.59–2.93), memory disturbance (HR 1.41; CI 1.26–1.59) and headache (HR 1.39; CI 1.31–1.47). Disturbance of smell and taste (HR 15.17; CI 12.23–18.83) and hair loss (HR 1.92; CI 1.66–2.22) was also more frequent in the exposed, but both groups had very low frequencies of registration before the pandemic. The likelihood for sleep disturbance, anxiety and depression were lower among exposed compared to the unexposed. However, the prevalence of anxiety and depression increased in both groups from 2019 to the follow-up period.

### Risk factors for outcome symptoms

Fatigue, memory disturbance and shortness of breath are outcome symptoms that represent different organ systems or possible mechanisms for post-COVID-19 symptoms, and these were used in analyses of demographic factors and prior illness as risk factors.

**Fig. 3:**
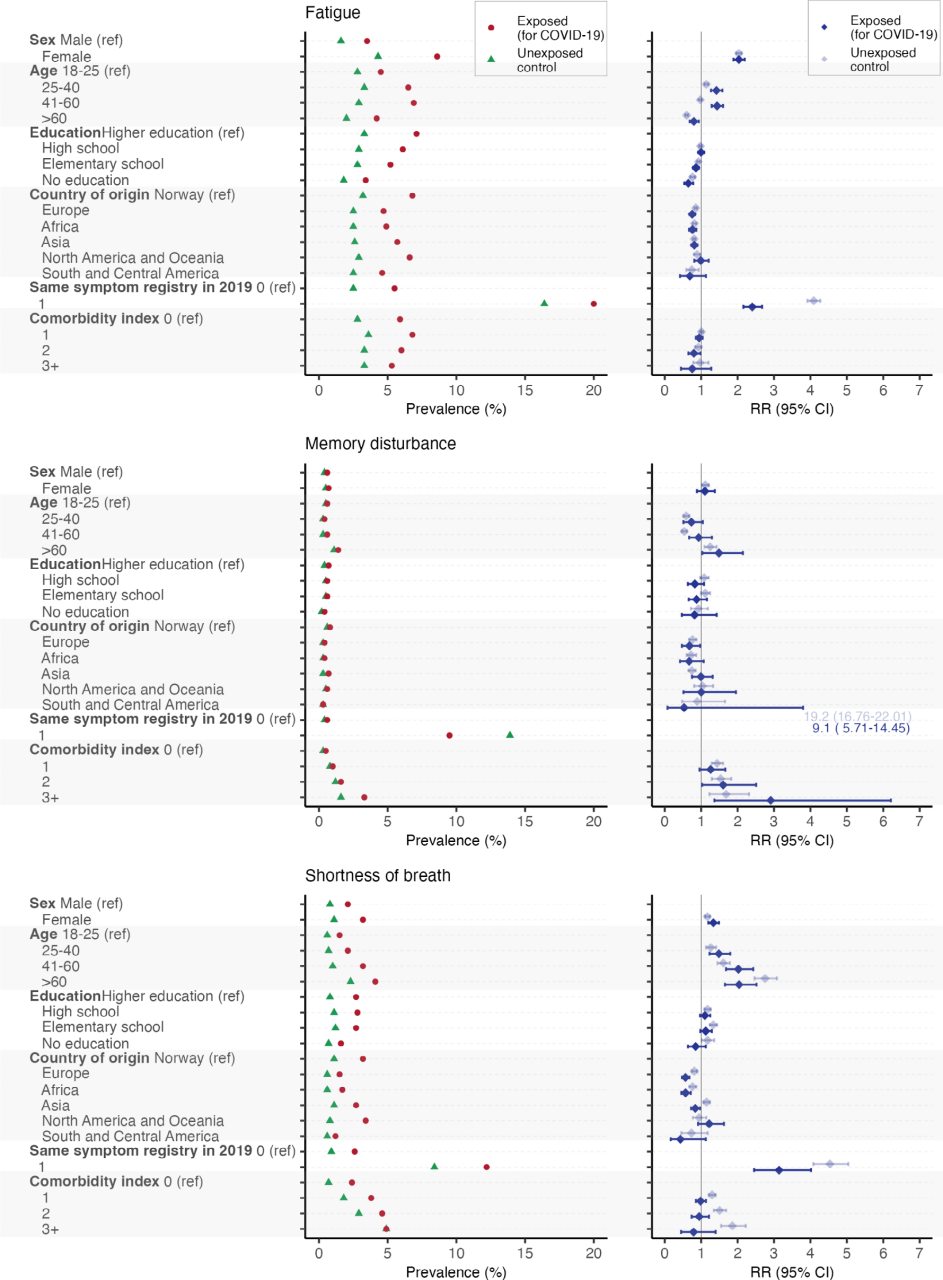
Prevalence and relative risks for fatigue, memory disturbance and shortness of breath among exposed and unexposed. Prevalence (left panels) and relative risks (RR) (right panels) for fatigue (upper panels), memory disturbance (middle panels), and shortness of breath (lower panels) diagnosed in general practice in Norway 3 to 12 months after a positive SARS-CoV-2 PCR test for the exposed or sampling date for the unexposed controls by potential risk conditions. RRs were calculated within the group of exposed and the group of unexposed separately

In Fig. 3 (left panels) we show the crude prevalence for these outcome symptoms in each stratum of the risk factors both among those exposed to COVID-19 and the unexposed control group. For fatigue we found higher prevalence in the exposed group for all strata of risk factors. Among those with pre-pandemic fatigue, the prevalence was high among unexposed but even higher among exposed. For memory disturbance the prevalence was highest among those with three or more comorbidities, and in this stratum the COVID-19 group had even higher prevalence than the control group. In contrast to fatigue, persons unexposed to COVID-19 with a pre-pandemic registration of memory disturbance had a higher post-pandemic prevalence of the same symptom than the unexposed group. Regarding shortness of breath there was a rather uniform pattern with higher prevalence of the symptom among those exposed to COVID-19 in all strata of risk factors, except no difference for those with three or more comorbidities.

To assess whether association with risk factors varied between the exposed COVID-19 group and the unexposed control group, we did stratified analyses of adjusted relative risks (RRs) for these two groups. These results are shown in Supplementary file Tables S2, S3, S4 and are the basis for Fig. 3 (right panels).

Sex was a risk factor for fatigue and shortness of breath both for exposed and unexposed (Fig. 3, right panels) with higher RR among females compared to males. We found a higher RR for fatigue in the COVID-19 group for age groups 25 to 60 years, but a lower RR among those over 60 years, compared to the unexposed. For memory disturbance the RR differed between exposed and non-exposed only in the age group 41–60, where it was higher in the COVID-19 group. Contrary, for shortness of breath the RR where lower in the age group > 60 among exposed compared to unexposed.

We found a slightly lower RR for fatigue among those with the lowest educational level. For shortness of breath the RRs were lower in the exposed group among those with lowest education, using highest education as reference.

There was a lower risk for outcome symptoms in both exposed and unexposed among those with country of origin from Europe, Africa and South and Central America, compared to Norway, but we found no systematic differences between exposed or unexposed.

Having an outcome symptom in the pre-pandemic period was a clear predictor of the respective outcome symptom after the pandemic in both exposed and unexposed groups, but with higher RRs in the unexposed, also for memory disturbance where the unexposed had higher absolute risk.

The number of comorbidities was not related to risk for fatigue. In the case of memory disturbance, the relative risk (RR) increased with the number of comorbidities and appeared to be higher in the COVID-19 group for those with three or more comorbidities. However, the difference did not reach statistical significance in the interaction analyses.

Conversely, for shortness of breath a higher number of comorbidities was associated with an increased RR for this symptom among the unexposed group, but this association was not observed among the exposed group.

The differences in impact of the risk factors were supported by interaction analyses (Supplementary file, Table S2, S3, S4, right columns).

Hospitalisation for COVID-19 was analysed as risk factor in the multivariate models for the exposed group (Supplementary file Table S5). We found that initial hospitalisation increased the risk moderately for fatigue (RR 1.61; CI 1.41–1.84) and memory disturbance (RR 1.58; CI 1.10–2.26) but was a marked risk for shortness of breath (RR 2.32; CI 1.98–2.73) in the post-COVID-19 period, as compared with the non-hospitalised COVID-19 group.

## Discussion

In this Norwegian nationwide registry-based study of primary care patients we found that the risk of fatigue or shortness of breath were more than doubled 3 to 12 months after COVID-19 compared to a control group of unexposed persons. The likelihood for memory disturbance, other respiratory symptoms, pain in abdomen, chest and musculoskeletal system and headache were also significantly higher in the COVID-19 group.

The risk of fatigue, memory disturbance and shortness of breath varied with sex, age, education and country of origin among patients after COVID-19. These risk factors generally acted in the same way in the unexposed control group, but pre-pandemic registration of comorbidities was less associated with shortness of breath after COVID-19 compared to unexposed. Having had the same symptom before the pandemic increased the risk for fatigue, memory disturbance and shortness of breath after COVID-19, but the associations were weaker in the exposed than in the unexposed group. Yet, a higher prevalence of outcome symptoms among the exposed support the hypothesis that such symptoms may be attributed to COVID-19, also in a low-risk primary care population. Post-COVID-19 symptoms were more frequent among those who were hospitalised during the acute infection compared to COVID-19 patients who were not hospitalised.

### Strengths and limitations

This is a nationwide registry-based study including all persons with a positive PCR test for SARS-CoV-2 and all contacts in a GP list patient system for the whole population, ruling out potential bias from selection or loss to follow up. The diagnoses were recorded by GPs in a “business as usual” situation, which strengthens the generalizability of the findings.

Another strength was the comprehensive data of the chosen outcome symptoms, comorbidities, and GP utilisation before the pandemic.

Most of the included COVID-19 patients in this study were unvaccinated since vaccination started in Norway at the turn of the year 2020/2021. At the end of our inclusion period (20 February 2021), only 7.5% of the Norwegian population had had a first dose and 3.7% of the population had two doses vaccinated [21] of whom many were nursing homes residents not included in our study population, 85 years of age or older, or health care workers. Consequently, the present study can be seen as a baseline study regarding post-COVID-19 symptoms in a predominantly unvaccinated population.

A limitation of our study is that for GPs, it is common to register only one or two ICPC-2 diagnosis codes, while patients may have presented several problems in one consultation. This is likely to limit the number of symptoms that we identified from registered codes, resulting in underestimation of the symptom burden. Since the data are from GP consultations which do not capture all symptoms in the population, the present study cannot be interpreted as prevalence study of post-COVID-19 symptoms in the general population.

Also, under-registration of COVID-19 is likely, as not all infected persons were tested. This was particular the case in the first 2–3 months of the pandemic due to a shortage of test kits. However, during the first phase in 2020 the incidence of COVID-19 in Norway was low due to lockdowns. This is supported by a study among 27 700 randomly selected persons at the end of 2020 detecting antibodies against SARS-CoV-2 only among 0.9% of the sample, which is close to the prevalence in the population with a positive PCR test at that time at 0.8% [22]. Therefore, in the whole population without positive PCR test the undiagnosed cases represent a very low share and should have neglectable effect on the results in the current study.

Having had COVID-19 may alter the utilisation of healthcare, but according to a Norwegian study the increased use of GP services related to COVID-19 gradually decreased to a normal level during the first three months after COVID-19 [23].

The lack of a specific diagnostic code for post-COVID-19 makes it difficult to assess the epidemiology of post-COVID-19 health issues. Walker et al. [24] found little use of codes for post-COVID-19 introduced for British GPs and recommended more awareness on coding of post-COVID-19 symptoms to increase possibilities for research and care planning. The current study is based on diagnoses irrespectively of the GPs’ interpretation of a possible connection to the prior COVID-19.

Still, those who have undergone COVID-19 may be more alert to symptoms reported as post-COVID-19 and present them to their GP. GPs may also have altered their coding practice, being more attentive to and record symptoms that could be related to prior COVID-19. This could result in confirmation and detection bias in our study. If so, the differences between exposed and unexposed may be overestimated in the current study. However, this is a challenge in all studies on registered symptoms that may be related to a certain disease.

The number of individuals with positive PCR test for SARS-CoV-2 was not very high during the exposure period used in this study. This leads to a small number of individuals in some of the risk factor strata within the COVID-19 group, and this is also reflected in the wide confidence intervals for some RRs in Fig. 3. The findings regarding these risk factors should be interpreted cautiously.

### Comparison with literature

#### Prevalence

Determining the prevalence of post-COVID-19 symptoms in the population is methodologically challenging, including confirmation of infection (self-report, health care reports or registries) and approaches to define post-COVID-19.

The prevalence of symptoms reported in meta-analyses published early during the pandemic were generally much higher than our findings [12, 18, 19]. Not all patients seek a GP for symptoms and the GP reported diagnoses do not reflect all symptoms experienced by the patients. When an early meta-analysis reports a prevalence of fatigue of 23 to 60%, compared to 6% in the present study, this may reflect different patient populations. A strength of our study is that it is based in primary care and therefore closer to true population rates than hospital-based studies. A later study has indicated lower prevalence, with 6% reporting post-COVID-19 symptoms after three months and about 1% after 12 months, which is more line with our data [6]. Further, a Norwegian cohort study among young adults found no association between serological signs of COVID-19 and symptoms 6 months later [25], using the broad WHO definition for post-COVID [8]. However, they showed a trend for increased prevalence of postinfectious fatigue, in line with our findings of a highest frequency and HR for this outcome symptom.

Unlike previous studies, particularly those relying on self-reported symptoms [18, 19], we found a lower likelihood for psychological symptoms in the post-COVID-19 period. This could indicate that these symptoms were frequently mild and not perceived as a reason to consult a GP, or it is possible that GPs chose to code other symptoms during the visit. On the other hand, as shown by others, we found an increase in prevalence of anxiety and depression also unexposed, possibly related to a more general effect of the pandemic [26]. It is worth noting that registry-based studies tend to report a lower prevalence of mild mental health problems compared to self-reported surveys [27].

A Dutch study, using data from a large population-based cohort study initiated before the pandemic, was able to establish a control group like we did [28]. The diagnostic categories are not quite comparable to the GP diagnostic codes used in our study, but the findings are similar to ours. Also, a study on non-hospitalised patients from UK showed hazard ratio for fatigue, shortness of breath and chest pain in the post-COVID-19 period in line with our findings [4]. In a recent study from Norway based on SARS-CoV-2 positive cases at the turn of year 2021/2022 there was marked lower HR for most symptoms [3], with HR for fatigue of 1.24 and 1.29 for Delta and Omicron respectively compared to the HR of 2.1 in our study. That study reported HRs of 1.29 (Delta) and 1.69 (Omicron) for shortness of breath compared to 2.8 in our study. These differences can be explained by a shorter observation period, maximum 4 months as compared to 12, and different covariates, as we included pre-pandemic health problems. However, this may also indicate a decrease in risk for post-COVID-19 symptom later in the pandemic, and an effect of vaccination of most of the population at that stage.

Recently, based on 9764 tested persons in the US, an attempt was made to develop a definition on post-acute sequelae of SARS-CoV-2 infection (PASC) [29]. However, only around 1300 were unexposed and symptoms were self-reported. It was acknowledged that developing a better definition would require analysis of prospectively and uniformly collected data from diverse uninfected and infected individuals. Our study may add knowledge to this field using a large sample of both exposed and unexposed.

In general, after various infections a minority of patients experience long term post-acute health problems [10]. Such symptoms are common across different infectious diseases, suggesting some common underlying mechanisms that, however, are poorly understood. In light of this knowledge, a post-acute syndrome following COVID-19 is not surprising nor exceptional.

#### Risk factors

Female sex, higher age, belonging to an ethnic minority group and a high disease burden prior to infection are documented risk factors for post-COVID syndrome [4, 5, 13, 30]. We found that the risk factors for fatigue, memory disturbance and shortness of breath were rather similar in exposed and unexposed. However, some minor divergences were found, as belonging to the age group 25 to 60 years was a stronger risk factor for fatigue in the exposed compared to the unexposed group.

In the literature, comorbid conditions are found to increase the risk for post-COVID-19 symptoms [4, 5]. However, using a score for comorbidities, we found no increase in relative risk for fatigue or shortness of breath with increasing number of comorbid conditions among exposed and a slight increase in risk for memory disturbance with three or more comorbidities. For shortness of breath, we found a lower relative risk with increasing numbers of comorbidities after COVID-19 compared to unexposed. This could indicate shortness of breath to be part of post-COVID-19 syndrome unrelated to previous diseases since the infection affects the respiratory system, whereas among the unexposed the risk increased with increasing comorbidity score. On the other hand, in our study the diagnosis shortness of breath might not have been registered if other diagnosed conditions were considered to explain the symptom by the GP, and this may more often be the case with lung symptom compared to fatigue and memory disturbance.

Outcome symptoms such as fatigue, memory disturbance and shortness of breath are common in the general population, and having had the same symptom before the pandemic markedly increased the risk for having an outcome symptom in the observation period. In clinical practice it is difficult or impossible to determine in each case whether a symptom actually was present prior to the infection, whether it is caused or aggravated by the infection or whether it appears by chance. This underlines the challenge using “post-COVID-19” as diagnosis in primary care, as shown by low-frequent use of such a code when introduced in the UK [24] and also indicate that a composite explanatory model is necessary to understand the mechanisms underlying post-COVID-19 symptoms [10, 25].

## Conclusion

In this study of a predominantly unvaccinated population, we found a higher prevalence of commonly defined post-COVID-19 symptoms 3 to 12 months after acute COVID-19 compared to an unexposed control group. This was particularly evident for fatigue and shortness of breath. The sociodemographic risk factors for outcome symptoms were generally similar for the exposed and the unexposed control group. However, having had a symptom previously represented a lower relative risk for a new encounter with the same symptom in the exposed COVID-19 group compared to the unexposed. This indicates that COVID-19 itself inflicted a stronger risk for a symptom in this group. In conclusion, COVID-19 was associated with a range of symptoms lasting more than three months after the infection.

### Electronic supplementary material

Below is the link to the electronic supplementary material.

Supplementary Material 1

## Data Availability

The data from registries used during the current study is not public available and permissions were given for use to this study only. Data can be delivered from the national health registries upon request after applying for ethical approval and permission to use data from the authorities responsible for the respective registries. Further information can be given by corresponding author.
