# Supplementary material for: Prevalence and predictors of post-COVID-19 symptoms in general practice - a registry-based nationwide study
Source: BMC Infect Dis. 2023 Oct 25;23:721. doi: 10.1186/s12879-023-08727-6 (PMC10599052; doi:10.1186/s12879-023-08727-6)
Supplement: Supplementary file 1 — Supplementary Material 1 [file 12879_2023_8727_MOESM1_ESM.docx]

**Supplementary file for:**

**“Prevalence and predictors of post-COVID-19 symptoms in general practice - a registry-based nationwide study.”**

**Supplementary table S1** shows the list of chronic conditions with the frequency of each of these conditions in the exposed and unexposed groups based on diagnoses registered by GPs in 2019. These conditions are summarised and used as a score in analyses.

**Supplementary tables S2a, S3a and S4a** show the number and percentage of persons with and without the outcome symptom within the COVID-19 group (exposed) and in the unexposed control group, respectively, for each of the considered risk factors presented in Figure 3.

**Supplementary tables S2b, S3b and S4b** show relative risks (RRs) and 95% CI for each risk factor for exposed and unexposed groups, separately. Univariable models include only the risk factor as an explanatory variable while multivariable models include all the risk factors listed in these tables and the number of pre-pandemic consultations.

Additionally, we present results (P-values) from interaction models including both exposed and unexposed to test the interaction between a risk factor and COVID-19 exposure. For each risk factor, a model including all the other risk factors and the interaction between the risk factor itself and exposure was used. P-values were calculated using cluster robust standard errors. Since P-values were not adjusted for multiple testing we consider a P-value < 0.001 as significant in these analyses. These P-values are shown in the right column in tables S2b, S3b and S4b.

**Supplementary table S5** shows RR and 95% CI for each risk factor in multivariable models adjusted for hospitalization, for the exposed group presented for fatigue, memory disturbance and shortness of breath in separate models.

| **Chronic condition** | **Included ICPC-2 codes** | **Exposed** | | **Unexposed** | |
| --- | --- | --- | --- | --- | --- |
|  |  | **N** | **%** | **N** | **%** |
| n |  | 53846 |  | 485757 |  |
| Hypertension | K86, K87 | 2631 | 4.9 | 23644 | 4.9 |
| Ischemic coronary disease | K74, K75, K76 | 427 | 0.8 | 4343 | 0.9 |
| Stroke | K89, K90 | 170 | 0.3 | 1809 | 0.4 |
| Atrial fibrillation | K78 | 350 | 0.7 | 3158 | 0.7 |
| Heart failure | K77 | 116 | 0.2 | 1283 | 0.3 |
| Diabetes | T89, T90 | 1833 | 3.4 | 14754 | 3.0 |
| Rheumatic disease | L88 | 310 | 0.6 | 3068 | 0.6 |
| Asthma | R96 | 1226 | 2.3 | 9810 | 2.0 |
| Chronic obstructive lung disease | R95 | 279 | 0.5 | 3247 | 0.7 |
| Cancer | B72, B73, B74, D74, D75, D76, D77, N74, R84, R85, U75, U76, U77, X75, X76, X77, Y77, Y78 | 494 | 0.9 | 4839 | 1.0 |
| Chronic Kidney/liver disease | U88, D72, D97 | 187 | 0.3 | 1804 | 0.4 |
| Multiple sclerosis | N86 | 67 | 0.1 | 822 | 0.2 |
| Depression | P76 | 1954 | 3.6 | 18990 | 3.9 |
| Anxiety and stress disorders | P75, P75, P82 | 1005 | 1.9 | 10228 | 2.1 |
| Addiction | P15, P18, P19 | 388 | 0.7 | 3879 | 0.8 |

**Supplementary table S1.** Frequencies of chronic conditions in the COVID-19 group (exposed) and unexposed group as registered by GPs in 2019

**Supplementary Table S2a.** **Outcome symptom: Fatigue.**

Number and proportion of risk factors for exposed and unexposed groups

| variable | value | Exposed | | | | Unexposed | | | |
| --- | --- | --- | --- | --- | --- | --- | --- | --- | --- |
|  |  | With symptom | | Without symptom | | With symptom | | Without symptom | |
|  |  | n | percent | n | percent | n | percent | n | percent |
| Sex | Male (ref.) | 979 | 3.5 | 26,821 | 96.5 | 4,105 | 1.6 | 245,940 | 98.4 |
|  | Female | 2,247 | 8.6 | 23,799 | 91.4 | 10,101 | 4.3 | 225,611 | 95.7 |
| Age | <25 (ref) | 433 | 4.5 | 9,165 | 95.5 | 2,293 | 2.8 | 78,923 | 97.2 |
|  | 25-40 | 1,227 | 6.5 | 17,639 | 93.5 | 5,721 | 3.3 | 166,011 | 96.7 |
|  | 41-60 | 1,280 | 6.9 | 17,355 | 93.1 | 4,841 | 2.9 | 160,209 | 97.1 |
|  | >60 | 286 | 4.2 | 6,461 | 95.8 | 1,351 | 2.0 | 66,408 | 98.0 |
| Education | Higher education (ref.) | 1,276 | 7.1 | 16,798 | 92.9 | 5,304 | 3.3 | 156,605 | 96.7 |
|  | No education | 120 | 3.4 | 3,411 | 96.6 | 546 | 1.8 | 29,884 | 98.2 |
|  | Primary | 788 | 5.2 | 14,497 | 94.8 | 3,832 | 2.8 | 133,862 | 97.2 |
|  | High school | 1,042 | 6.1 | 15,914 | 93.9 | 4,524 | 2.9 | 151,200 | 97.1 |
| Origin country | Norway (ref.) | 1,816 | 6.8 | 24,904 | 93.2 | 8,248 | 3.2 | 245,806 | 96.8 |
|  | Africa | 267 | 4.9 | 5,178 | 95.1 | 1,063 | 2.5 | 40,639 | 97.5 |
|  | Asia | 558 | 5.7 | 9,248 | 94.3 | 2,132 | 2.6 | 79,762 | 97.4 |
|  | Europe | 476 | 4.7 | 9,649 | 95.3 | 2,310 | 2.5 | 89,698 | 97.5 |
|  | North America and Oceania | 94 | 6.6 | 1,332 | 93.4 | 382 | 2.9 | 12,829 | 97.1 |
|  | South and Central America | 15 | 4.6 | 309 | 95.4 | 71 | 2.5 | 2,817 | 97.5 |
| Number of comorbidities | 0 (ref.) | 2,607 | 5.9 | 41,867 | 94.1 | 11,189 | 2.8 | 388,434 | 97.2 |
|  | 1 | 514 | 6.8 | 7,073 | 93.2 | 2,470 | 3.6 | 67,102 | 96.4 |
|  | 2 | 92 | 6.0 | 1,448 | 94.0 | 463 | 3.3 | 13,531 | 96.7 |
|  | 3+ | 13 | 5.3 | 232 | 94.7 | 84 | 3.3 | 2,484 | 96.7 |
| Number of pre-pandemic consultations | 0 (ref.) | 670 | 3.5 | 18,406 | 96.5 | 2,693 | 1.5 | 182,893 | 98.5 |
|  | 1-2 | 1,003 | 5.9 | 16,009 | 94.1 | 4,281 | 2.9 | 144,596 | 97.1 |
|  | 3-5 | 851 | 8.0 | 9,766 | 92.0 | 3,699 | 4.1 | 86,858 | 95.9 |
|  | 5+ | 702 | 9.8 | 6,439 | 90.2 | 3,533 | 5.8 | 57,204 | 94.2 |
| Pre-pandemic fatigue | 0 (ref.) | 2,889 | 5.5 | 49,271 | 94.5 | 11,740 | 2.5 | 458,946 | 97.5 |
|  | 1 | 337 | 20.0 | 1,349 | 80.0 | 2,466 | 16.4 | 12,605 | 83.6 |

**Supplementary Table S2b.** **Outcome symptom: Fatigue**.

Univariable and multivariable regression results for risk factors. Modified Poisson regression is used to calculate RR within exposed and unexposed groups, separately.

| variable | value | Exposed | | | | Unexposed | | | | Interaction** |
| --- | --- | --- | --- | --- | --- | --- | --- | --- | --- | --- |
|  |  | Univariable | | Multivariable* | | Univariable | | Multivariable* | |  |
|  |  | RR | CI | RR | CI | RR | CI | RR | CI | p |
| Sex  (ref. male) | Female | 2.45 | 2.28-2.64 | 2.04 | 1.89-2.2 | 2.61 | 2.52-2.71 | 2.03 | 1.96-2.11 | 0.0951 |
| Age  (ref. <25) | 25-40 | 1.44 | 1.3-1.6 | 1.42 | 1.27-1.58 | 1.18 | 1.12-1.24 | 1.14 | 1.08-1.2 | 0.0006 |
|  | 41-60 | 1.52 | 1.37-1.69 | 1.44 | 1.29-1.6 | 1.04 | 0.99-1.09 | 0.98 | 0.93-1.03 | 0.0000 |
|  | >60 | 0.94 | 0.81-1.09 | 0.80 | 0.69-0.93 | 0.71 | 0.66-0.75 | 0.60 | 0.56-0.64 | 0.0006 |
| Education  (ref. higher education) | No education | 0.48 | 0.4-0.58 | 0.65 | 0.54-0.78 | 0.55 | 0.5-0.6 | 0.76 | 0.7-0.84 | 0.0875 |
|  | Primary | 0.73 | 0.67-0.8 | 0.86 | 0.78-0.94 | 0.85 | 0.82-0.89 | 0.92 | 0.88-0.96 | 0.0015 |
|  | Highschool | 0.87 | 0.8-0.94 | 1.00 | 0.92-1.08 | 0.89 | 0.85-0.92 | 0.98 | 0.94-1.02 | 0.5924 |
| Origin country  (ref. Norway) | Europe | 0.69 | 0.63-0.76 | 0.75 | 0.68-0.83 | 0.77 | 0.74-0.81 | 0.85 | 0.81-0.89 | 0.0236 |
|  | Africa | 0.72 | 0.64-0.82 | 0.76 | 0.67-0.87 | 0.79 | 0.74-0.84 | 0.81 | 0.76-0.87 | 0.0945 |
|  | Asia | 0.84 | 0.76-0.92 | 0.81 | 0.74-0.89 | 0.80 | 0.77-0.84 | 0.81 | 0.77-0.85 | 0.4216 |
|  | North America and Oceania | 0.97 | 0.79-1.19 | 0.99 | 0.81-1.21 | 0.89 | 0.81-0.99 | 0.89 | 0.8-0.98 | 0.4921 |
|  | South and Central America | 0.68 | 0.41-1.12 | 0.69 | 0.42-1.13 | 0.76 | 0.6-0.95 | 0.74 | 0.59-0.93 | 0.7574 |
| Number of comorbidities  (ref. 0) | 1 | 1.16 | 1.05-1.27 | 0.94 | 0.86-1.04 | 1.27 | 1.21-1.32 | 1.01 | 0.96-1.05 | 0.2006 |
|  | 2 | 1.02 | 0.83-1.25 | 0.80 | 0.65-0.98 | 1.18 | 1.08-1.29 | 0.92 | 0.84-1.01 | 0.1618 |
|  | 3+ | 0.91 | 0.53-1.54 | 0.75 | 0.45-1.28 | 1.17 | 0.95-1.44 | 0.97 | 0.78-1.2 | 0.3869 |
| Pre-pandemic fatigue  (ref. no symptom) | 1 | 3.61 | 3.26-4 | 2.40 | 2.16-2.67 | 6.56 | 6.3-6.83 | 4.09 | 3.92-4.27 | 0.0000 |

*Models include all the variables listed on the table and adjusted for the number of pre-pandemic consultations.

**Significance level of an interaction term testing the interaction between the variable (risk factor) and COVID-19 exposure based on multivariable models constructed with all the listed variables and the number of pre-pandemic consultations. Only one interaction term was used in the models, which resulted in 6 models for the 6 variables. P-values presented are calculated by using cluster robust standard errors and are not adjusted for multiple testing.

Supplementary Table S3a. **Outcome symptom: Memory disturbance.**

Number and proportion of risk factors for exposed and unexposed groups

| variable | value | Exposed | | | | Unexposed | | | |
| --- | --- | --- | --- | --- | --- | --- | --- | --- | --- |
|  |  | With symptom | | Without symptom | | With symptom | | Without symptom | |
|  |  | n | percent | n | percent | n | percent | n | percent |
| Sex | Male (ref.) | 153 | 0.6 | 27,647 | 99.4 | 981 | 0.4 | 249,064 | 99.6 |
|  | Female | 188 | 0.7 | 25,858 | 99.3 | 1,190 | 0.5 | 234,522 | 99.5 |
| Age | <25 (ref) | 54 | 0.6 | 9,544 | 99.4 | 427 | 0.5 | 80,789 | 99.5 |
|  | 25-40 | 81 | 0.4 | 18,785 | 99.6 | 512 | 0.3 | 171,220 | 99.7 |
|  | 41-60 | 114 | 0.6 | 18,521 | 99.4 | 510 | 0.3 | 164,540 | 99.7 |
|  | >60 | 92 | 1.4 | 6,655 | 98.6 | 722 | 1.1 | 67,037 | 98.9 |
| Education | Higher education (ref.) | 120 | 0.7 | 17,954 | 99.3 | 587 | 0.4 | 161,322 | 99.6 |
|  | No education | 15 | 0.4 | 3,516 | 99.6 | 73 | 0.2 | 30,357 | 99.8 |
|  | Primary | 99 | 0.6 | 15,186 | 99.4 | 718 | 0.5 | 136,976 | 99.5 |
|  | High school | 107 | 0.6 | 16,849 | 99.4 | 793 | 0.5 | 154,931 | 99.5 |
| Origin country | Norway (ref.) | 205 | 0.8 | 26,515 | 99.2 | 1,430 | 0.6 | 252,624 | 99.4 |
|  | Africa | 22 | 0.4 | 5,423 | 99.6 | 128 | 0.3 | 41,574 | 99.7 |
|  | Asia | 66 | 0.7 | 9,740 | 99.3 | 273 | 0.3 | 81,621 | 99.7 |
|  | Europe | 38 | 0.4 | 10,087 | 99.6 | 268 | 0.3 | 91,740 | 99.7 |
|  | North America and Oceania | 9 | 0.6 | 1,417 | 99.4 | 62 | 0.5 | 13,149 | 99.5 |
|  | South and Central America | 1 | 0.3 | 323 | 99.7 | 10 | 0.3 | 2,878 | 99.7 |
| Number of comorbidities | 0 (ref.) | 229 | 0.5 | 44,245 | 99.5 | 1,374 | 0.3 | 398,249 | 99.7 |
|  | 1 | 79 | 1.0 | 7,508 | 99.0 | 585 | 0.8 | 68,987 | 99.2 |
|  | 2 | 25 | 1.6 | 1,515 | 98.4 | 170 | 1.2 | 13,824 | 98.8 |
|  | 3+ | 8 | 3.3 | 237 | 96.7 | 42 | 1.6 | 2,526 | 98.4 |
| Number of pre-pandemic consultations | 0 (ref.) | 55 | 0.3 | 19,021 | 99.7 | 387 | 0.2 | 185,199 | 99.8 |
|  | 1-2 | 102 | 0.6 | 16,910 | 99.4 | 609 | 0.4 | 148,268 | 99.6 |
|  | 3-5 | 98 | 0.9 | 10,519 | 99.1 | 569 | 0.6 | 89,988 | 99.4 |
|  | 5+ | 86 | 1.2 | 7,055 | 98.8 | 606 | 1.0 | 60,131 | 99.0 |
| Pre-pandemic memory disturbance | 0 (ref.) | 323 | 0.6 | 53,334 | 99.4 | 1,947 | 0.4 | 482,193 | 99.6 |
|  | 1 | 18 | 9.5 | 171 | 90.5 | 224 | 13.9 | 1,393 | 86.1 |

**Supplementary Table S3b.** **Outcome symptom: Memory disturbance**.

Univariable and multivariable regression results for risk factors. Modified Poisson regression is used to calculate RR within exposed and unexposed groups, separately.

| variable | value | Exposed | | | | Unexposed | | | | Interaction** |
| --- | --- | --- | --- | --- | --- | --- | --- | --- | --- | --- |
|  |  | Univariable | | Multivariable* | | Univariable | | Multivariable* | |  |
|  |  | RR | CI | RR | CI | RR | CI | RR | CI | p |
| Sex  (ref. male) | Female | 1.31 | 1.06-1.62 | 1.10 | 0.88-1.37 | 1.29 | 1.18-1.4 | 1.11 | 1.02-1.21 | 0.9584 |
| Age  (ref. <25) | 25-40 | 0.76 | 0.54-1.08 | 0.73 | 0.51-1.05 | 0.57 | 0.5-0.64 | 0.59 | 0.51-0.67 | 0.1025 |
|  | 41-60 | 1.09 | 0.79-1.5 | 0.93 | 0.67-1.3 | 0.59 | 0.52-0.67 | 0.53 | 0.47-0.61 | 0.0007 |
|  | >60 | 2.42 | 1.73-3.39 | 1.49 | 1.03-2.15 | 2.03 | 1.8-2.28 | 1.25 | 1.09-1.42 | 0.5228 |
| Education  (ref. higher education) | No education | 0.64 | 0.37-1.09 | 0.82 | 0.47-1.43 | 0.66 | 0.52-0.84 | 0.92 | 0.72-1.18 | 0.7135 |
|  | Primary | 0.98 | 0.75-1.27 | 0.87 | 0.66-1.16 | 1.44 | 1.29-1.6 | 1.11 | 0.99-1.25 | 0.0369 |
|  | Highschool | 0.95 | 0.73-1.23 | 0.83 | 0.63-1.08 | 1.40 | 1.26-1.56 | 1.08 | 0.97-1.21 | 0.0179 |
| Origin country  (ref. Norway) | Europe | 0.49 | 0.35-0.69 | 0.68 | 0.47-0.97 | 0.52 | 0.45-0.59 | 0.76 | 0.67-0.87 | 0.7310 |
|  | Africa | 0.53 | 0.34-0.82 | 0.67 | 0.42-1.07 | 0.55 | 0.46-0.65 | 0.72 | 0.6-0.87 | 0.7539 |
|  | Asia | 0.88 | 0.67-1.16 | 0.99 | 0.75-1.32 | 0.59 | 0.52-0.67 | 0.74 | 0.65-0.85 | 0.0454 |
|  | North America and Oceania | 0.82 | 0.42-1.6 | 1.00 | 0.51-1.95 | 0.83 | 0.65-1.07 | 1.04 | 0.81-1.33 | 0.9604 |
|  | South and Central America | 0.40 | 0.06-2.88 | 0.53 | 0.07-3.8 | 0.62 | 0.33-1.14 | 0.89 | 0.48-1.66 | 0.6898 |
| Number of comorbidities  (ref. 0) | 1 | 2.02 | 1.57-2.61 | 1.26 | 0.95-1.66 | 2.45 | 2.22-2.69 | 1.44 | 1.29-1.6 | 0.2713 |
|  | 2 | 3.15 | 2.09-4.75 | 1.60 | 1.02-2.51 | 3.53 | 3.02-4.14 | 1.53 | 1.29-1.83 | 0.8167 |
|  | 3+ | 6.34 | 3.16-12.72 | 2.91 | 1.36-6.21 | 4.76 | 3.51-6.45 | 1.68 | 1.22-2.32 | 0.3218 |
| Pre-pandemic memory disturbance  (ref. no symptom) | 1 | 15.82 | 10.04-24.94 | 9.09 | 5.71-14.45 | 34.45 | 30.26-39.21 | 19.20 | 16.76-22.01 | 0.0007 |

*Models include all the variables listed on the table and are adjusted for the number of pre-pandemic consultations.

**Significance level of an interaction term testing the interaction between the variable (risk factor) and COVID-19 exposure based on multivariable models constructed with all the listed variables and the number of pre-pandemic consultations. Only one interaction term was used in the models, which resulted in 6 models for the 6 variables. P-values presented are calculated by using cluster robust standard errors and are not adjusted for multiple testing.

**Supplementary Table S4a. Outcome symptom: Shortness of breath.**

Number and proportion of risk factors for exposed and unexposed group

| variable | value | Exposed | | | | Unexposed | | | |
| --- | --- | --- | --- | --- | --- | --- | --- | --- | --- |
|  |  | With symptom | | Without symptom | | With symptom | | Without symptom | |
|  |  | n | percent | n | percent | n | percent | n | percent |
| Sex | Male (ref.) | 582 | 2.1 | 27,218 | 97.9 | 2,097 | 0.8 | 247,948 | 99.2 |
|  | Female | 844 | 3.2 | 25,202 | 96.8 | 2,663 | 1.1 | 233,049 | 98.9 |
| Age | <25 (ref) | 146 | 1.5 | 9,452 | 98.5 | 459 | 0.6 | 80,757 | 99.4 |
|  | 25-40 | 403 | 2.1 | 18,463 | 97.9 | 1,164 | 0.7 | 170,568 | 99.3 |
|  | 41-60 | 599 | 3.2 | 18,036 | 96.8 | 1,597 | 1.0 | 163,453 | 99.0 |
|  | >60 | 278 | 4.1 | 6,469 | 95.9 | 1,540 | 2.3 | 66,219 | 97.7 |
| Education | Higher education (ref.) | 479 | 2.7 | 17,595 | 97.3 | 1,276 | 0.8 | 160,633 | 99.2 |
|  | No education | 56 | 1.6 | 3,475 | 98.4 | 219 | 0.7 | 30,211 | 99.3 |
|  | Primary | 408 | 2.7 | 14,877 | 97.3 | 1,588 | 1.2 | 136,106 | 98.8 |
|  | High school | 483 | 2.8 | 16,473 | 97.2 | 1,677 | 1.1 | 154,047 | 98.9 |
| Origin country | Norway (ref.) | 866 | 3.2 | 25,854 | 96.8 | 2,906 | 1.1 | 251,148 | 98.9 |
|  | Africa | 91 | 1.7 | 5,354 | 98.3 | 271 | 0.6 | 41,431 | 99.4 |
|  | Asia | 263 | 2.7 | 9,543 | 97.3 | 881 | 1.1 | 81,013 | 98.9 |
|  | Europe | 154 | 1.5 | 9,971 | 98.5 | 580 | 0.6 | 91,428 | 99.4 |
|  | North America and Oceania | 48 | 3.4 | 1,378 | 96.6 | 105 | 0.8 | 13,106 | 99.2 |
|  | South and Central America | 4 | 1.2 | 320 | 98.8 | 17 | 0.6 | 2,871 | 99.4 |
| Number of comorbidities | 0 (ref.) | 1,057 | 2.4 | 43,417 | 97.6 | 2,971 | 0.7 | 396,652 | 99.3 |
|  | 1 | 286 | 3.8 | 7,301 | 96.2 | 1,253 | 1.8 | 68,319 | 98.2 |
|  | 2 | 71 | 4.6 | 1,469 | 95.4 | 409 | 2.9 | 13,585 | 97.1 |
|  | 3+ | 12 | 4.9 | 233 | 95.1 | 127 | 4.9 | 2,441 | 95.1 |
| Number of pre-pandemic consultations | 0 (ref.) | 273 | 1.4 | 18,803 | 98.6 | 790 | 0.4 | 184,796 | 99.6 |
|  | 1-2 | 427 | 2.5 | 16,585 | 97.5 | 1,346 | 0.9 | 147,531 | 99.1 |
|  | 3-5 | 365 | 3.4 | 10,252 | 96.6 | 1,179 | 1.3 | 89,378 | 98.7 |
|  | 5+ | 361 | 5.1 | 6,780 | 94.9 | 1,445 | 2.4 | 59,292 | 97.6 |
| Pre-pandemic shortness of breath | 0 (ref.) | 1,362 | 2.6 | 51,959 | 97.4 | 4,378 | 0.9 | 476,826 | 99.1 |
|  | 1 | 64 | 12.2 | 461 | 87.8 | 382 | 8.4 | 4,171 | 91.6 |

**Supplementary Table S4b.** **Outcome symptom: Shortness of breath.**

Univariable and multivariable regression results for risk factors. Modified Poisson regression is used to calculate RR within exposed and unexposed groups, separately.

| variable | value | Exposed | | | | Unexposed | | | | Interaction** |
| --- | --- | --- | --- | --- | --- | --- | --- | --- | --- | --- |
|  |  | Univariable | | Multivariable* | | Univariable | | Multivariable* | |  |
|  |  | RR | CI | RR | CI | RR | CI | RR | CI | p |
| Sex  (ref. male) | Female | 1.55 | 1.39-1.72 | 1.33 | 1.2-1.49 | 1.35 | 1.27-1.43 | 1.17 | 1.1-1.24 | 0.0217 |
| Age  (ref. <25) | 25-40 | 1.40 | 1.16-1.69 | 1.49 | 1.23-1.8 | 1.20 | 1.08-1.34 | 1.26 | 1.13-1.41 | 0.1896 |
|  | 41-60 | 2.11 | 1.77-2.53 | 2.02 | 1.68-2.43 | 1.71 | 1.54-1.9 | 1.61 | 1.45-1.79 | 0.0882 |
|  | >60 | 2.71 | 2.22-3.3 | 2.04 | 1.65-2.52 | 4.02 | 3.63-4.46 | 2.75 | 2.46-3.08 | 0.0002 |
| Education  (ref. higher education) | No education | 0.60 | 0.45-0.79 | 0.85 | 0.64-1.13 | 0.91 | 0.79-1.05 | 1.17 | 1.01-1.36 | 0.0006 |
|  | Primary | 1.01 | 0.88-1.15 | 1.12 | 0.98-1.29 | 1.46 | 1.36-1.57 | 1.33 | 1.23-1.44 | 0.0000 |
|  | Highschool | 1.07 | 0.95-1.22 | 1.10 | 0.97-1.25 | 1.37 | 1.27-1.47 | 1.17 | 1.09-1.26 | 0.0072 |
| Origin country  (ref. Norway) | Europe | 0.47 | 0.4-0.56 | 0.57 | 0.48-0.68 | 0.55 | 0.5-0.6 | 0.81 | 0.74-0.89 | 0.0354 |
|  | Africa | 0.52 | 0.42-0.64 | 0.57 | 0.46-0.71 | 0.57 | 0.5-0.64 | 0.76 | 0.67-0.87 | 0.1119 |
|  | Asia | 0.83 | 0.72-0.95 | 0.84 | 0.72-0.97 | 0.94 | 0.87-1.01 | 1.15 | 1.06-1.24 | 0.0013 |
|  | North America and Oceania | 1.04 | 0.78-1.38 | 1.22 | 0.91-1.63 | 0.69 | 0.57-0.84 | 0.94 | 0.77-1.14 | 0.0285 |
|  | South and Central America | 0.38 | 0.14-1.01 | 0.43 | 0.16-1.13 | 0.51 | 0.32-0.83 | 0.73 | 0.45-1.18 | 0.4936 |
| Number of comorbidities  (ref. 0) | 1 | 1.59 | 1.4-1.8 | 0.98 | 0.86-1.13 | 2.42 | 2.27-2.59 | 1.30 | 1.21-1.4 | 0.0000 |
|  | 2 | 1.94 | 1.53-2.45 | 0.95 | 0.74-1.21 | 3.93 | 3.55-4.35 | 1.51 | 1.35-1.69 | 0.0000 |
|  | 3+ | 2.06 | 1.18-3.6 | 0.79 | 0.45-1.4 | 6.65 | 5.59-7.91 | 1.86 | 1.55-2.23 | 0.0000 |
| Pre-pandemic shortness of breath  (ref. no symptom) | 1 | 4.77 | 3.77-6.04 | 3.14 | 2.45-4.02 | 9.22 | 8.34-10.2 | 4.53 | 4.08-5.04 | 0.0000 |

*Models include all the variables listed on the table and adjusted for number of pre-pandemic consultations.

**Significance level of an interaction term testing the interaction between the variable (risk factor) and COVID-19 exposure based on multivariable models constructed with all the listed variables and the number of pre-pandemic consultations. Only one interaction term was used in the models, which resulted in 6 models for the 6 variables. P-values presented are calculated by using cluster robust standard errors and are not adjusted for multiple testing.

**Supplementary Table S5**.

Multivariable regression results for risk factors for **exposed** – adjusted for hospitalization.

| variable | value | **Fatigue** | | **Memory disturbance** | | **Shortness of breath** | |  |
| --- | --- | --- | --- | --- | --- | --- | --- | --- |
|  |  | RR | CI | RR | CI | RR | CI |  |
| Sex (ref. male) | Female | 2.05 | 1.9-2.21 | 1.11 | 0.89-1.38 | 1.35 | 1.22-1.51 |  |
| Age (ref. <25) | 25-40 | 1.41 | 1.26-1.57 | 0.73 | 0.51-1.04 | 1.46 | 1.21-1.77 |  |
|  | 41-60 | 1.41 | 1.26-1.57 | 0.91 | 0.65-1.27 | 1.92 | 1.6-2.31 |  |
|  | >60 | 0.74 | 0.63-0.86 | 1.36 | 0.94-1.97 | 1.71 | 1.38-2.12 |  |
| Education (ref. higher education) | No education | 0.64 | 0.53-0.78 | 0.81 | 0.46-1.41 | 0.83 | 0.63-1.11 |  |
|  | Primary | 0.85 | 0.78-0.93 | 0.87 | 0.65-1.15 | 1.11 | 0.97-1.28 |  |
|  | Highschool | 1.00 | 0.92-1.08 | 0.82 | 0.63-1.07 | 1.09 | 0.96-1.24 |  |
| Origin country (ref. Norway) | Europe | 0.76 | 0.68-0.83 | 0.68 | 0.47-0.98 | 0.57 | 0.48-0.68 |  |
|  | Africa | 0.75 | 0.66-0.86 | 0.67 | 0.42-1.06 | 0.56 | 0.45-0.7 |  |
|  | Asia | 0.80 | 0.73-0.88 | 0.98 | 0.74-1.3 | 0.81 | 0.7-0.94 |  |
|  | North America and Oceania | 0.99 | 0.81-1.2 | 1.00 | 0.51-1.95 | 1.21 | 0.91-1.61 |  |
|  | South and Central America | 0.68 | 0.42-1.12 | 0.53 | 0.07-3.79 | 0.43 | 0.16-1.12 |  |
| Number of comorbidities (ref. 0) | 1 | 0.93 | 0.84-1.03 | 1.24 | 0.94-1.64 | 0.95 | 0.83-1.09 |  |
|  | 2 | 0.78 | 0.63-0.96 | 1.57 | 1-2.46 | 0.90 | 0.7-1.16 |  |
|  | 3+ | 0.71 | 0.42-1.21 | 2.80 | 1.31-5.98 | 0.71 | 0.4-1.27 |  |
| Pre-pandemic fatigue  (ref. no symptom) | 1 | 2.39 | 2.15-2.66 | 8.85 | 5.52-14.18 | 2.97 | 2.33-3.8 |  |
| Hospitalization  (ref. no hospitalization) | 1 | 1.61 | 1.41-1.84 | 1.58 | 1.1-2.26 | 2.32 | 1.98-2.73 |  |
